# Antibiotic Residues in Raw Cow’s Milk: A Systematic Review of the Last Decade

**DOI:** 10.3390/foods13233758

**Published:** 2024-11-24

**Authors:** Lucyana Vieira Costa, Clarice Gebara, Ozana de Fátima Zacaroni, Natylane Eufransino Freitas, Adriele Nascimento da Silva, Cristiano Sales Prado, Iolanda Aparecida Nunes, Valéria Quintana Cavicchioli, Francine Oliveira Souza Duarte, Moacir Evandro Lage, Fabiane Rodrigues de Alencar, Bruna Aparecida Souza Machado, Katharine Valéria Saraiva Hodel, Cíntia Minafra

**Affiliations:** 1 Universidade Estadual de Goiás, Campus Sul Ipameri, Ipameri 75780-000, Goiás, Brazil; 2 Centro de Pesquisa em Alimentos, Escola de Veterinária e Zootecnia, Campus Samambaia da Universidade Federal de Goiás, Goiânia 74690-900, Goiás, Brazil; claricegebara@ufg.br (C.G.); natyllane@hotmail.com (N.E.F.); adriele.silva@labcpa.com.br (A.N.d.S.); cristiano_prado@ufg.br (C.S.P.); iolanda_nunes@ufg.br (I.A.N.); valeria.cavicchioli@ufg.br (V.Q.C.); francineoliveira@ufg.br (F.O.S.D.); moacirlage@ufg.br (M.E.L.); 3 Departamento de Zootecnia, Escola de Veterinária e Zootecnia, Campus Samambaia da Universidade Federal de Goiás, Goiânia 74690-900, Goiás, Brazil; ozacaroni@ufg.br; 4 Escola de Agronomia, Campus Samambaia da Universidade Federal de Goiás, Goiânia 74690-900, Goiás, Brazil; fabianerodrigues@discente.ufg.br; 5 SENAI Instituto de Inovação (ISI) Sistemas Avançados em Saúde (CIMATEC ISI SAS), SENAI CIMATEC, Centro Universitário, Salvador 41650-010, Bahia, Brazil; brunam@fieb.org.br (B.A.S.M.); katharine.hodel@fieb.org.br (K.V.S.H.)

**Keywords:** analysis, food safety, milk quality, One Health

## Abstract

The inappropriate use of antimicrobials in dairy animals can lead to residues in raw milk and in dairy products. Foods containing residues of this nature, whether in the short, medium, or long term, cause serious health harm. Absence of these compounds in foods should be a premise for declaring safety. This systematic review aimed to identify the antibiotic residues most frequently found in raw bovine milk and the methodologies used to detect such residues over the ten years from 2013 to 2023. PRISMA guidelines for systematic reviews were followed, by searching the Web of Science, PubMed Central, Scopus, and Springer databases. The search strategy identified 248 articles, and after applying the selection and quality assessment criteria, 16 studies were selected. The number of samples analyzed was 411,530, of which 0.21% tested positive for some type of antibiotic. Eight classes and 38 different types of antibiotics were identified. The most common class was tetracycline, with emphasis on sulfonamides and quinolones, which have shown increasing prevalence among residues in milk in recent years. A total of 56.25% of the studies employed rapid kits to detect residues, 18.75% chromatography, and 25% both techniques. Antibiotic residues in bovine raw milk should be a great concern for animal, environmental, and human health.

## 1. Introduction

Bovine milk, which has high nutritional value, is one of the most consumed foods globally and plays a major role in the global economy [1]. Due to its balanced composition of proteins, fats, and carbohydrates, and its status as a comparatively inexpensive and widely available animal-origin food, it is essential for human nutrition [2]. Milk production involves around 150 million families worldwide and, in most developing countries, occurs on small farms. This not only helps families remain in the countryside but also contributes to food security [3], as it provides access to this nutritious and important food for various community sections.

In animal production, antibacterial substances are used as therapeutic agents against various pathogenic microorganisms [4] and can also be used in metaphylaxis protocols [5]. The use of antibiotics in animal production continues to be a relevant issue, requiring constant monitoring by authorities that inspect products of animal origin in order to guarantee food safety [6]. Regulations such as Maximum Residue Limits (MRLs) and the Codex Alimentarius Commission have been developed to determine withdrawal periods after antimicrobial therapy and to ensure the correct handling and marketing of milk [7,8].

The ingestion of uncontrolled amounts of antibacterial substances can cause several health changes, triggering allergies, hypersensitivity, aplastic anemia, alterations in gastrointestinal microflora, and the selection of resistant intestinal bacteria. Moreover, it can cause financial losses to dairy industries, as these residues inhibit the growth of crops necessary for producing certain dairy products [9,10].

Rolli et al. [11] observed that the total disposal, without use for human or animal consumption, of milk from treated cows at risk of antibiotic residues represents a 22% loss relative to the total cost per animal during the lactation period, a factor that impacts milk production. These costs refer mainly to losses of raw milk not intended for processing and to improvement of dairy products.

In another spectrum, the term “One Health” can be widely explored for the minimization of risk to human and animal health [12]. This approach emphasizes animal welfare and the rational use of antimicrobials [13,14] and the safety of milk, including the absence of pathogens and contaminants [15]. Thus, this systematic review aims to survey studies conducted worldwide in the last ten years (2013–2023), identify the most frequent antibiotic residues in raw milk, and outline the methodologies used to detect these residues.

## 2. Materials and Methods

This review adhered to the guidelines of Preferred Reporting Items for Systematic Reviews and Meta-Analyses (PRISMA) [16], Open Science Framework registration—Identifier: DOI 10.17605/OSF.IO/3ZMJC. The research team determined the proposal, along with its specific objectives and inclusion and exclusion criteria. To avoid bias, the searches were conducted independently by the authors Lucyana Vieira Costa (LVC) and Cíntia Minafra (CM), and the results from both searches were identical.

### 2.1. Search Strategy and Selection Criteria

The data sources for this review included electronic data in English from the Web of Science, PubMed Central, Scopus, and Springer databases. No language restrictions were applied, and the search across all databases was conducted on 7 September 2023. The search formulation was defined as follows: “raw milk” AND (“cow” OR “bovine”) AND (“antibiotic residue” OR “antimicrobial residue”), and it included articles from the last ten years (2013–2023).

### 2.2. Inclusion and Exclusion Criteria

**Figure 1:**
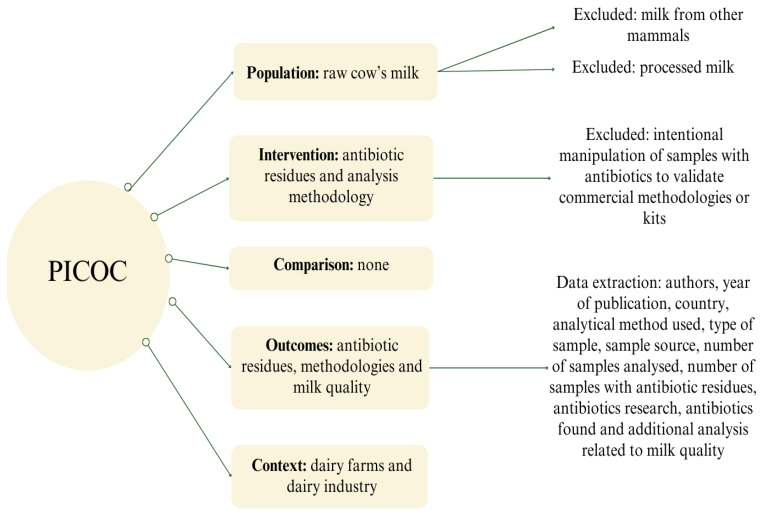
Selection criteria based on PICOC (population, intervention, comparison, outcome, and context) and data extracted from the results generated.

The study design was based on PICOC (population, intervention, comparison, outcome, and context). The selected population for this review was raw cow’s milk; therefore, articles that did not focus on bovine milk or involved processed milk were excluded. The intervention analyzed was the presence of antibiotic residues in milk and the methodologies used to detect such contaminants. Studies that intentionally manipulated samples with antibiotics to validate methodologies or commercial kits, or to confirm the period of milk discard after antibiotic treatment, were excluded from the research. No comparison was made with the proposed intervention. The results included data on the most commonly found antibiotic classes, the most commonly applied analysis techniques or commercial kits, and additional analyses that evaluated some aspect of milk quality (when performed). The context was set in dairy farms and the dairy industry. Figure 1 presents a summary of these steps.

Finally, studies that were not available in full, that lacked detailed information on antibiotic residues in raw milk, or that did not provide specific results for raw milk, in the form of less substantive review articles, book chapters, dissertations, or theses, were also excluded.

### 2.3. Data Extraction and Analysis

The data required according to the research objectives were extracted from the articles after the final selection by the aforementioned two authors (LVC and CM). This data included the names of the authors, year of publication, country, analytical method(s) used, type of sample used (refrigerated or frozen), source of the sample, number of samples analyzed, quantity of samples with antibiotic residues, antibiotics investigated or validated, antibiotics found, and additional analyses related to milk quality, when present (Figure 1).

### 2.4. Quality Assessment

A quality assessment was applied to the selected articles. This review consisted of three questions aimed at selecting the articles that best fit the scope of this review. The first question pertained to the number of antibiotic residue classes identified in the study; the second addressed the use of rapid kits for detecting residues; and the third concerned whether additional analyses were conducted to assess the physical-chemical, compositional, and/or microbiological quality of the milk. Each question could receive a score of zero (0), half (0.5), or one (1.0), with each article potentially achieving a maximum of three points. Of the selected articles, only those articles that scored two (2.0) or higher in the quality assessment were definitively selected for inclusion in this review. Two reviewers, LVC and CM, critically assessed the quality of the studies, and any disagreements were resolved by consensus.

## 3. Results and Discussion

### 3.1. The Survey Process

**Figure 2:**
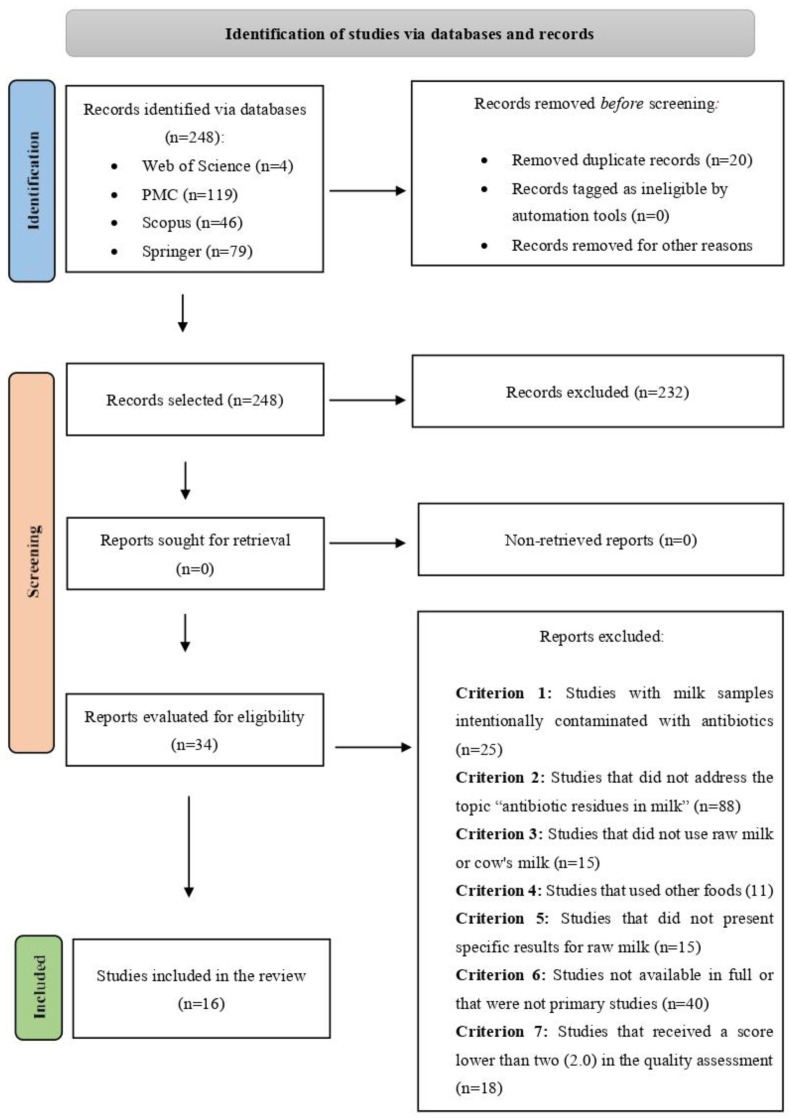
A flowchart summarizing the study selection process.

Initially, the search strategy identified a total of 248 articles. After removing duplicates, 228 articles remained for the initial selection phase. After evaluating the titles and abstracts, 88 articles were excluded for not addressing antibiotic residues in milk—most of these dealt with antibacterial resistance genes and mastitis—and another 35 were excluded because they were not primary studies. This left 105 studies eligible for full-text screening. Of these, 9 studies did not use raw milk, 6 did not use cow’s milk, and 11 focused on other animal products and were thus disqualified. The full texts of five articles were unavailable and could not be included. In 25 articles, there was manipulation of samples with intentional addition of antibiotics, and these were therefore excluded from the study. A total of 15 studies were excluded for not presenting specific data on raw milk, leaving 34 articles selected for quality assessment. Of these, 16 received a score of two (2.0) or higher and were included in the study, with their data subsequently extracted (Figure 2).

### 3.2. Data and Characteristics of the Included Studies

The data from the 16 final articles selected for this review are summarized in Table 1. Regarding the origins of the samples, 4 out of the 16 articles (25%) utilized samples from more than one source, including individual cows, refrigeration tanks, transport vehicles, dairy silos, street vendors, and others [17,18,19,20]. Both Orwa et al. [19] and Ondieki et al. [18] observed antibiotic residues across all examined sources—including cows, transporters, and storage silos. These data emphasize the necessity of conducting tests at different stages of dairy production, particularly individual assessments post-antibiotic treatment. Often, residues exceed permissible levels even after the designated withdrawal period has elapsed [21], which could lead to widespread residue distribution throughout the dairy supply chain.

**Figure 3:**
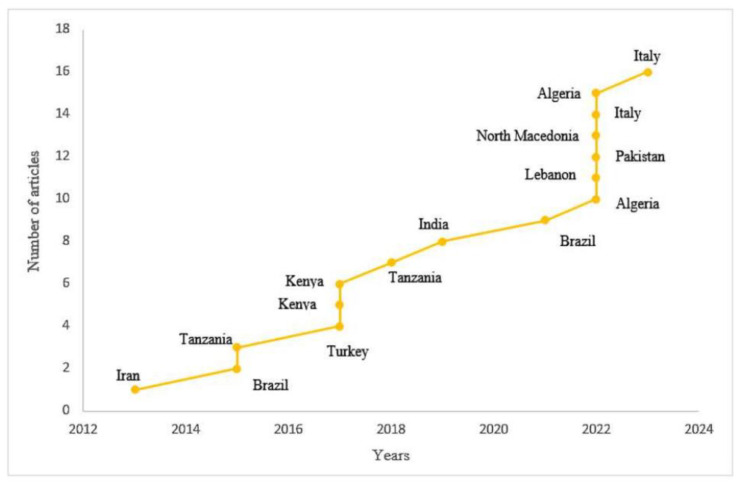
Distribution of publications by year and country.

During the specified article selection timeframe of 2013–2023, 10 of the 16 studies were published in the past five years, reflecting a growing focus on antibiotic residues in research, as depicted in Figure 3. These studies spanned four continents, with one additional study conducted in Eurasia (6.25%). Africa produced the highest number of selected articles (37.5%), followed by Asia (25%), Europe (18.75%), and the Americas (12.5%), with the latter consisting solely of research conducted in Brazil.

The data elucidate the global concern about the effects that antibiotic residues can have on human health, bacterial resistance, and the milk chain. This reiterates the findings of Sachi et al. [34], who observed that after the first detection of antibiotic residues in milk in 1960, there was an increasing trend in the number of publications on the topic, with a particularly sharp rise in detections after the year 2000.

According to the methodology defined for the selection of articles for this review, there was an absence of research carried out in the last ten years by the United States, China, Spain, and Germany, which are the countries that have historically published the most on this subject, according to Sachi et al. [34]. Particularly until 2015, Van Boeckel et al. [35] noted that countries with low or medium milk production, such as those in Africa, had limited data on the use of antibiotics and the residue levels of these products in animal-origin foods.

In this review, it was possible to observe a change in this scenario. In 2015, a study was conducted in Tanzania [17], which collected data on milk quality, including analysis of antibiotic residues, and awareness among parts of the dairy sector about the risks of consuming raw milk in the city of Arusha and in the Meru District Council. Following this, five more studies were published until July of this year (2023) in different African countries: Kenya in 2017 [18,19], Tanzania in 2018 [25], and Algeria in 2022 [28,32].

In the 16 studies analyzed, the samples were stored for later residue analysis following collection. The storage method depended on logistical needs; some samples were refrigerated or frozen for preservation. Specifically, 56.25% of the studies refrigerated their samples exclusively, 18.75% opted for freezing, and 12.50% required both methods. The remaining 12.50% of the articles did not specify their storage protocols. Butovskaya et al. [33] performed one of the studies that involved both refrigerating and freezing the samples. This was necessary because a large volume of samples was initially tested with the Delvotest kit, and a subset was later analyzed using high-performance liquid chromatography (HPLC), which required freezing.

In the literature, it is reported that storing milk under refrigerated conditions or after heat treatments can interfere with the results of antibiotic residue evaluation, as it has been proven that beta-lactam and tetracycline molecules are the most sensitive to these thermal procedures [36]. This highlights the importance of maintaining a short interval between the time of collection and the execution of the analysis, as well as adopting temperatures and storage conditions approved by official methods, always avoiding prolonged periods.

#### 3.2.1. Residual Antibiotic Rate

Among the 16 articles included, 13 studies (81.25%) reported the detection of antibiotic residues in samples. The lowest incidence was 0.09%, found in Italy [33], and the highest was 82.70% in Tanzania [25]. Three studies (18.75%) detected no residues in the analyzed raw milk samples [17,23,27]. Table 2 details the number of samples analyzed by these 16 studies and the count of positive samples.

Altogether, the 16 articles analyzed 411,530 samples, with 849 (0.21%) testing positive for antibiotic residues. Of the three studies with no residue findings, one was conducted in Tanzania, examining raw milk from small dairy farms, suppliers, and commercial retailers [17], and two were in Brazil, both analyzing milk from refrigerated tanks on dairy farms, conducted in 2015 [23] and 2021 [27], respectively.

In the methodology section, 43.75% of the articles (7 out of 16) did not specify the classes of antibiotics investigated. The remaining 56.25% (nine articles) not only specified the antibiotic classes, but all investigated beta-lactam residues. Among these, two studies (12.5%) exclusively searched for beta-lactams [24,33], and four studies (25%) looked for both beta-lactams and tetracyclines [27,28,29,32]. The other three studies expanded their search to include sulfonamides and aminoglycosides [19]; quinolones, sulfonamides, and amphenicols [26]; and quinolones and sulfonamides [20].

**Figure 4:**
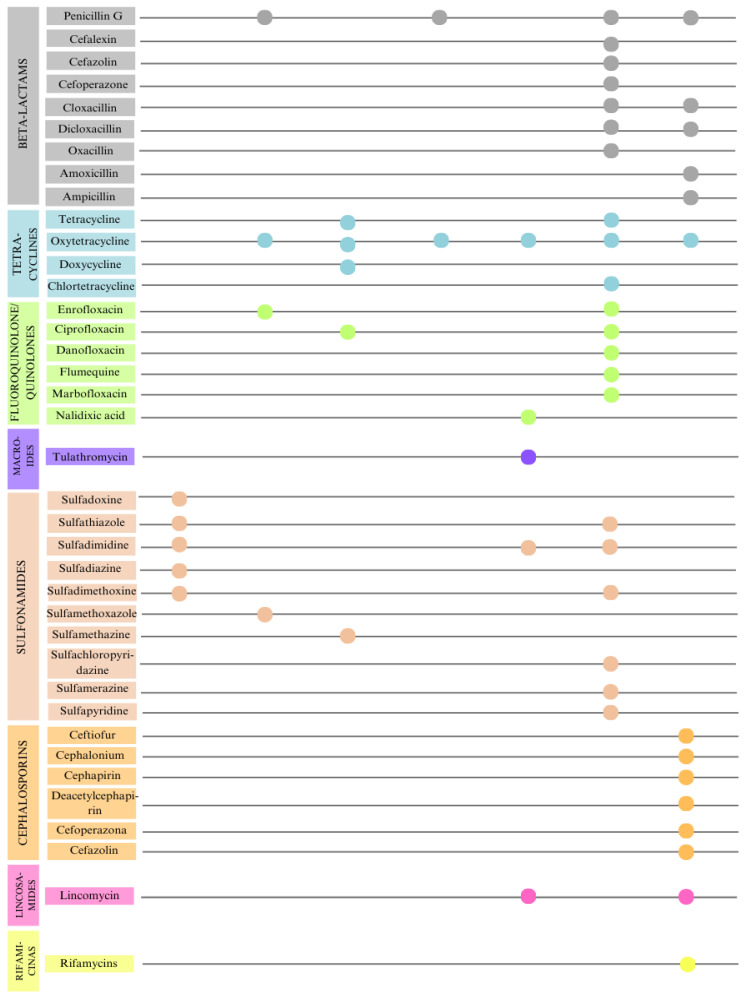
The classes of antibiotics and their respective compounds reported by 6 of the 16 studies included in this review, identified by chromatographic techniques or ELISA [19,20,26,29,31,32,33].

The chromatographic or ELISA (Enzyme Linked Immuno Sorbent Assay) techniques used in the studies identified eight classes and 38 different antibiotics (Figure 4). Results from commercial kits were not includes because they did not individually identify the antibiotics present. Tetracycline was the most frequently found group, appearing in 7 of the 16 articles (43.75%), followed by beta-lactams and sulfonamides, each found in 5 articles (31.25%), and quinolones in 4 (25%). A similar pattern was observed in a systematic review conducted in 2021 in China, where the most frequently evaluated residues over the last three decades were beta-lactams, such as penicillin and streptomycin, followed by tetracyclines, like tetracycline and oxytetracycline, and sulfonamides, such as sulfadimidine [37]. Historically, beta-lactams have been the antibiotics most commonly used to treat mastitis, primarily administered through intramammary infusion because of their wide range of antibacterial activity [38]. This widespread use accounts for their frequent detection in studies.

Seven studies (43.75%) assessed whether results were within the maximum residue limit (MRL) set by the European Union (EU) [8]; five of these reported levels exceeding the EU’s permitted levels [18,19,26,32,33]. The MRLs, established for each pharmacologically active substance, are designed as precautionary measures to safeguard human health. Nonetheless, reports suggest that even minimal short- to long-term consumption can induce adverse reactions in the human body [39].

Beta-lactams, commonly used in dairy farming, have been extensively studied. Meklati et al. [32] found cloxacillin concentrations ranging from 4.90 to 1505 µg/kg, up to 50 times the EU’s allowed limit, with 55.32% of samples (26 of 47) exceeding the MRL. Similarly, Butovskaya et al. [33] detected that 28% of milk samples from Italian farms contained beta-lactam residues surpassing the MRL. In addition, two samples contained rifaximin and ampicillin at levels five times higher than allowed: 324 μg/kg (rifamycin) and 28 μg/kg for (beta-lactam), respectively.

Moudgil et al. [26] identified 19 positive samples, 6 of which (31.58%) were above the MRL/EU. These included beta-lactams (two oxytetracycline, one penicillin G) and three fluoroquinolones (enrofloxacin), marking this class as the second most prevalent in their study. Similarly, Meklati et al. [32] reported flumequine in 42.30% of their samples, though only one exceeded the MRL (52 μg/kg), and found enrofloxacin in 15.40% of samples, with one sample above the MRL (100 μg/kg).

The increase in the use of quinolones and fluoroquinolones, a subclass of quinolones, has been observed in various countries, including Africa, in recent years [40]. The growth in the use of this class is primarily attributed to its low cost, easy availability, and broad spectrum of activity [41].

#### 3.2.2. Detection Methods

**Figure 5:**
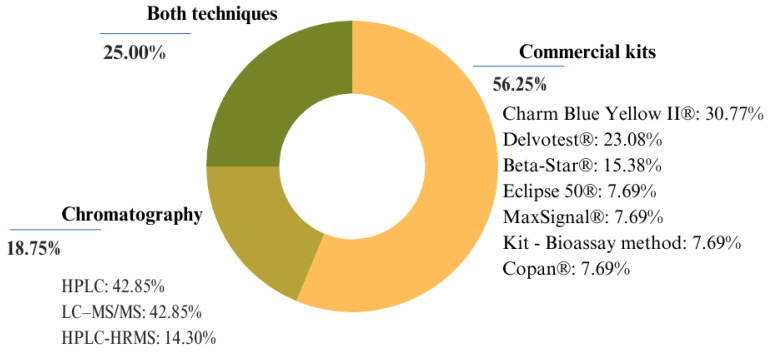
Methodologies used in the detection of antibiotic residues: distribution of chromatographic methods and commercial kits used in studies.

Of the 16 studies selected for this review, 9 (56.25%) used kits, 3 (18.75%) utilized chromatography, and 4 (25%) employed both techniques (Figure 5). The three most widely used commercial kits were Charm Blue Yellow II ^®^, Delvotest SP-NT ^®^, and BetaStar ^®^, each detecting specific classes of antibiotics but all capable of identifying beta-lactams and tetracyclines. The chromatography techniques included HPLC [19,29,33], LC-MS/MS (Liquid Chromatography Tandem Mass Spectrometry) [20,30,32], and HPLC-HRMS (High-Performance Liquid Chromatography coupled with High-Resolution Mass Spectrometry) [31], with one study using ELISA [26].

Chromatography techniques are favored for analyzing antibiotic residues in food due to their greater precision and accuracy [39]. Matrix calibration curves were obtained using antibiotic standards in blank milk samples. Dilutions were performed in the milk to obtain antibiotic concentration levels for constructing the calibration curve. In contrast, well-known commercial rapid-screening kits provide qualitative results and are less expensive, yielding good results and being simple to use [42]. Although chromatographic techniques are often used to verify the accuracy of these kits, none of the four studies that used both methods performed this comparison. Instead, kits were used for screening, and any positive findings were further analyzed by chromatography to identify the residues and their concentrations. This approach highlights that the rapid kits, validated and recognized by regulatory bodies, are extensively utilized across various countries.

Butovskaya et al. [33] utilized the Delvotest SP-NT ^®^ kit and noted its capability to detect rifaximin presence in samples, despite the absence of clear data on the test’s sensitivity to this compound. Bion et al. [43] reported that rifaximin residues are detectable at 60 μg/kg (MRL); however, in the study by Butovskaya et al. [33], rifaximin levels identified by LC-HRMS ranged from 11 to 17 μg/kg. The authors suggested that Delvotest SP-NT ^®^ might detect rifaximin due to its association with cefacetrile, a first-generation cephalosporin [44], although cefacetrile presence was not confirmed since it was not part of the compounds selected by the LC-HRMS method.

As mentioned previously, beta-lactams were the second most frequently identified class in this review and through LC-MS/MS analysis. Meklati et al. [32] found a total of 161 compounds from five antibiotic families—beta-lactams, tetracyclines, fluoroquinolones, sulfonamides, and diaminopyrimidines—in 52 samples, with beta-lactams (penicillin G, cloxacillin, dicloxacillin, oxacillin) and their metabolites as the most frequently detected residues. The authors employed the BetaStar^®^ kit for screening, and chromatographic results were consistent with the screening, confirming the test’s sensitivity to this antibiotic class.

In cases of ambiguous results from rapid tests, retesting or chromatographic analysis is recommended to confirm the results. Butovskaya et al. [33] followed this protocol, reanalyzing 21 uncertain samples from the Delvotest SP-NT^®^ test with LC-HRMS and finding that only 6 (28.57%) contained detectable beta-lactam residues. Conversely, Orwa [19] achieved 97.1% efficiency using the Charm Blue Yellow II^®^ test, with only 9 of 309 samples failing to yield definitive results.

Biosensors have emerged as another method for analyzing antibiotic residues in milk in recent years. Recognized for their simplicity, on-site application, cost–low-cost, and high specificity [45], biosensors offer a viable alternative to more sensitive but also demanding techniques like chromatography and ELISA, which require greater time and investment. Within this review, one study employing the bioassay method (Kit ID YRM1007-401) identified 41 positive samples, accounting for 20.5% of the 200 samples analyzed [29].

Regardless of the analytical method—chromatography, ELISA, or commercial kits for rapid screening—it is suggested that regulatory and administrative agencies use a variety of reliable, accurate, and recognized methods for control purposes [46]. This approach ensures more reliable results and, consequently, greater safety for consumers.

#### 3.2.3. Composition, Physicochemical, and Microbiological Analyses

Criteria such as somatic cell count, physical parameters, compositional profile, and microbiological profile, as well as their interaction, are vital elements in defining raw milk quality. Of the selected studies, 62.5% (10/16) conducted one or more analyses to evaluate milk quality. Table 3 describes the analyses performed in these articles.

In five articles (31.25%), a combination of analyses was employed, spanning physicochemical, microbiological, and/or centesimal composition methods. In four articles (25%), the analyses were limited to physicochemical and composition tests. One article (6.25%) conducted exclusively microbiological analyses [20], totaling six articles that performed microbiological evaluations, e.g., total bacterial count, coliform, yeast, and mold counts, and other assessments.

One of the reasons for including additional analyses in the quality criteria of selected articles is the known interference of certain factors with the results of antibiotic residue tests, such as pH variations and high somatic cell count (SCC) [42]. These additional assessments related to milk quality are crucial.

Mahmoudi et al. [22] found a significant correlation between SCC and the presence of antibiotic residues, with winter milk samples showing 32.50% more contamination compared to summer samples (25%). The study noted a strong statistical correlation (r^2^ = 0.305) between high SCC and antibiotic residues, with winter and summer means of 1052.00 ± 321.26 and 890.71 ± 250.73 cells/mL, respectively.

In contrast, studies by Ürkek et al. [24] and Sora et al. [31] also included SCC analyses but did not report any correlation with antibiotic residues. Similarly, Angicano et al. [23] and Oliveira et al. [27] found no residues in their samples, making it impossible to establish any correlation despite performing SCC analysis.

#### 3.2.4. Risk and Effects of Antibiotic Residues

For microbiological assessments, Joubrane [20] evaluated 195 milk samples in Lebanon, finding significant non-compliance with national and international standards. Regarding aerobic mesophilic bacteria, 96% of samples did not meet standards; 81% for total coliforms; 17% for *Staphylococcus aureus*; 35% for *Salmonella* spp.; and 65% for *Escherichia coli*. Although residues of oxytetracycline, tetracycline, ciprofloxacin, sulfamethazine, and doxycycline were below the MRL, resistance was noted among *E. coli* isolates to tetracycline (41%), doxycycline (39%), nalidixic acid (20%), and ciprofloxacin (3%). *Staphylococcus aureus* isolates showed resistance to doxycycline (63%) and tetracycline (51%), and *Salmonella* spp. isolates were resistant to doxycycline (12%), tetracycline (6%), and nalidixic acid (4%).

Consumption of milk containing antibiotic residues above the MRL set by the European Union (EU) and Codex Alimentarius Commission can cause allergies, toxicity, and other adverse reactions in consumers. These residues also contribute to the growing problem of antimicrobial resistance, a significant public health concern [47]. Thus, precise and rigorous monitoring of the levels of antibiotic residues and other factors that might interfere with product quality is warranted. It is imperative to use validated and precise analytical methods, whether commercial kits or laboratory analyses, to ensure reliable results for producers, the industry, and consumers.

## 4. Conclusions

There has been a marked increase in research on “antibiotic residues”, as evidenced by the publication of 10 out of the 16 articles in this review within the last five years. This surge is primarily attributed to concerns related to the One Health approach and the development of new analytical techniques, confirming the importance of the topic for the entire dairy industry, the general population, and researchers. Tetracycline was the most frequently detected class of antibiotics, with notable increases in sulfonamides and quinolones among milk residues. Global investment in this research area is essential to enhance control over antibiotic residues in milk, as has been demonstrated in Africa, which contributed 37.50% of the articles in this review. Further studies should explore viable field-applicable detection techniques and expand the use of commercial kits, which were employed in over 50% of the studies. This approach will enable more reliable and accurate data collection, considering that methods like chromatography are costly, labor-intensive, and impractical for field use.

## Figures and Tables

**Table 1:** General data from the selected studies.

Authorship, Year	Country of Origin of the Study	Type of Sample Analyzed	Sample Source
Mahmoudi et al. [22], 2013	Iran	Unspecified *	Dairy farms (unspecified *)
Ngasala et al. [17], 2015	Tanzania	Refrigerated raw milk	Small dairy farms (unspecified *), milk suppliers and resellers
Angicano [23], 2015	Brazil	Refrigerated raw milk	Cooling tank
Ürkek et al. [24], 2017	Turkey	Refrigerated raw milk	Cooling tanks (9 conventional and 9 organic farms)
Ondieki [18], 2017	Kenya	Refrigerated raw milk	Dairy farms (unspecified *) and milk suppliers
Orwa et al. [19], 2017	Kenya	Refrigerated raw milk	Dairy farms (unspecified *), dairy farms (individual samples), conveyors, and silos.
Gwandu et al. [25], 2018	Tanzania	Frozen raw milk	Small dairy farms (unspecified *)
Moudgil et al. [26] 2019	India	Frozen raw milk	Individuals (cows)
Oliveira et al. [27], 2021	Brazil	Refrigerated raw milk	Cooling tanks
Zeghilet et al. [28], 2022	Argelia	Unspecified *	Milk collectors
Joubrane [20], 2022	Lebanon	Refrigerated raw milk	Small to large farms (unspecified *), collection centers, cooperatives and street vendors
Raza et al. [29], 2022	Pakistan	Refrigerated raw milk	Cooling tank
Hajrulai-Musliu et al. [30], 2022	North Macedonia	Refrigerated raw milk	Dairy farms (unspecified *)
Sora et al. [31], 2022	Italy	Frozen raw milk	Dairy farms (unspecified *)
Meklati et al. [32], 2022	Argelia	Frozen raw milk, Refrigerated raw milk	Dairy industries
Butovskaya et al. [33], 2023	Italy	Frozen raw milk, Refrigerated raw milk	Dairy farms (unspecified *)

* Unspecified: the study did not provide complete information.

**Table 2:** The sample number and positive results for antibiotic residues in the articles included in the study, regardless of the analysis method.

Author	Number of Samples	Positive Samples	Positive Samples (%)
Mahmoudi [22]	200	115	57.70
Ngasala [17]	35	0	0
Angicano et al. [23]	920	0	0
Ürkek et al. [24]	Conventional farms: 50; Organic farms: 47	Conventional farms: 1; Organic farms: 2	Conventional farms: 2.00; Organic farms: 4.23
Ondieki [18]	Farms: 207; Suppliers: 152	Farms: 32; Suppliers: 28	Farms: 15.50; Suppliers: 18.40
Orwa [19]	309	95	30.74
Gwandu et al. [25]	98	81	82.70
Moudgil et al. [26]	168	19	11.30
Oliveira et al. [27]	22	0	0
Zeghilet et al. [28]	109	11	10.09
Joubrane [20]	84	14	16.67
Raza et al. [29]	200	41	20.50
Hajrulai-Musliu et al. [30]	120	5	4.16
Sora et al. [31]	331	7	2.11
Meklati et al. [32]	Kit BetaStar^®^: 445; LC-MS/MS: 52 *	BetaStar^®^: 34; LC-MS/MS: 47 *	BetaStar^®^: 7.64; LC-MS/MS: 90.4 *
Butovskaya et al. [33]	Delvotest^®^ SP-NT: 408,033; HPLC: 100 *	Delvotest^®^ SP-NT: 364; HPLC: 54 *	Delvotest^®^ SP-NT: 0.09; HPLC: 54 *
Total	411,530	849	0.21

* Values already included in the total analyzed per kit.

**Table 3:** The composition, physicochemical, or microbiological analyses performed in conjunction with antibiotic residue analysis by 10 of the 16 studies included in the review.

Authorship	Physicochemical or Microbiological Analysis
Mahmoudi et al. [22]	Somatic cell count (SCC)
Ngasala et al. [17]	pH, acidity, density, and total viable count
Angicano et al. [23]	Fat, protein, casein, lactose, total solids, solids not-fat, freezing point, urea, and SCC
Ürkek et al. [24]	SCC, Total aerobic mesophilic bacteria count, coliforms, yeasts, molds, and coagulase-positive *S. aureus*
Ondieki et al. [18]	Added water, fat, solids not-fat, protein, specific gravity, and freezing point
Gwandu et al. [25]	pH, acidity, density, ash, fat, total solids, total viable count, and total coliforms
Moudgil et al. [26]	Mycotoxins (aflatoxin M1 and ochratoxin A)
Oliveira et al. [27]	SCC, standard plate count (SPC), microbiome, psychrotrophic microorganism count, and differential bacterial count
Joubrane et al. [20]	Count of total aerobic mesophilic bacteria, total coliforms, *E. coli*, *S. aureus*, *L. monocytogenes*, *Salmonella* spp., and b-hemolytic streptococci
Sora et al. [31]	SCC, fat, protein, extraction, identification, and quantification of *S. aureus*, *S. Agalactiae* and *M. Bovis*

## Data Availability

No new data were created or analyzed in this study. Data sharing is not applicable to this article.
